# Supplementary material for: Multifunctional 3D-Printable Photocurable Elastomer with Self-Healing Capability Derived from Waste Cooking Oil
Source: Molecules. 2025 Apr 18;30(8):1824. doi: 10.3390/molecules30081824 (PMC12029562; doi:10.3390/molecules30081824)
Supplement: Supplementary file 1 [file molecules-30-01824-s001.zip › molecules-3553545-supplementary.pdf]

Number of pages: 19

Number of tables: 6

Number of figures: 10

Number of Video: 4

List

Table S1. Fatty acid compositions (wt.%) of WCO. Three main types of fatty acids: saturated (C n:0), monounsaturated (C n:1), and polyunsaturated with two or three double bonds (C n:2/3).

Table S2. The Lovibond color codes of WCO, WFAEE and WEFAEE.

Table S3. The viscosities of liquid WMFAEE-HPA and other control sample at room temperature.

Table S4. The thermogravimetric datas of WMFAEE-HPA elastomers.

Table S5. The testing results of initial adhesion of WMFAEE-HPA elastomers.

Table S6. The glass transition temperatures (T_g) of WMFAEE-HPA elastomers

Figure S1. Photograph of WCO, WFAEE and WEFAEE showing the difference in their colors.

Figure S2. The absorption spectrum of the WMFAEE-HPA elastomer (A4 sample, red) and the emission spectrum of the LCD curing light sources (blue).

Figure S3. The holding adhesion times of WMFAEE-HPA elastomers on steel surface with different amounts of HPA.

Figure S4. The stress-strain curves: (a) WMFAEE-HPA elastomers with varying HPA content; The pure HPA (A1) and EWOMA-HPA (A7) were both listed as control samples; (b) the A4 elastomer at different temperatures; (c) A4 elastomer before and after 24-hours self-healing; (d) A4 elastomer before and after 12-hours welding; (e) A4 elastomer before and after different reprocessing cycles.

Figure S5. The DTG curves of WMFAEE-HPA elastomers and pure HPA (A1 sample).

Figure S6. The DMA spectra of of WMFAEE-HPA elastomers and pure HPA (A1 sample).

Figure S7. The DSC curves of of WMFAEE-HPA elastomers and pure HPA (A1 sample): (a) A1, (b) A2, (c) A3, (d) A4, (e) A5, (f) A6.

Figure S8. CAD model diagram of 1BA dumbbell-shaped spline according to the China Standard GB/T 1040.2-2006.

Figure S9. Modelling of HPA and WMFAEE-HPA with Cu matrix as interface before self-healing.

Figure 10. The cohesive energy of HPA (a) and WMFAEE-HPA (b), of which the insets are the cohesive energy of the single chain.

Video S1. Demonstration of the deformation-recovery characteristics of a vertically suspended 4D-printed a4 elastomer dart under adhesion.

Video S2. Showcase of 4D-printed space sieves crafted from a4 elastomer, vertically mounted and suspended on glass surfaces.

Video S3. Presentation of repeated adhesion capabilities of a 4D-printed panda cut-out sticker composed of a4 elastomer.

Video S4. Room temperature recovery demonstration of a 4D-printed dart made from a4 elastomer.

Table S1. Fatty acid compositions (wt.%) of WCO. Three main types of fatty acids: saturated (C n:0), monounsaturated (C n:1), and polyunsaturated with two or three double bonds (C n:2/3).

Fatty acid		Fatty acid compositions (wt.%)
Caprylic	C 8:0	0.3
Myristic	C 14:0	1.0
Palmitic	C 16:0	11.1
Palmitoleic	C 16:1	1.9
Stearic	C 18:0	2.4
Oleic	C 18:1	46.1
Linoleic	C 18:2	30.5
Linolenic	C 18:3	2.9
Arachidic	C 20:0	2.1

Table S2. The Lovibond color codes of WCO, WFAEE and WEFAEE.

Sample	Y (Yellow)	R (Red)	B (Blue)	Light field	Dark field
WCO	20.2	3.9	0	0	0.1
WFAEE	10.1	5	0	0	0
WEFAEE	0.3	5.2	0	0	0.1

Table S3. The viscosities of liquid WMFAEE-HPA and other control sample at room temperature.

Sample	Viscosity (mPa·s)
Pure HPA (A1)	11
A2	14
A3	15
A4	20
A5	24
A6	28
WMFAEE	79

Table S4. The thermogravimetric datas of WMFAEE-HPA elastomers.

Sample	Initial decomposition temperature (℃)	The temperature at which the decomposition rate is maximum (℃)	Peak decomposition rate (%/min)	The final carbon residue (%)
A2	217.40	387.28	19.88	1.22
A3	195.47	388.69	8.01	1.99
A4	193.96	376.17	8.39	1.49
A5	192.58	383.58	8.81	1.58
A6	191.19	388.05	9.92	1.12

Table S5. The testing results of initial adhesion of WMFAEE-HPA elastomers.

Sample	Ball No.	Ball diameter (mm)
A2	2	1.59
A3	4	3.18
A4	5	3.97
A5	2	1.59
A6	2	1.59

Table S6. The glass transition temperatures (T_g) of WMFAEE-HPA elastomers

Sample	Glass transition temperature (T_g, ℃)
Pure HPA (A1)	19.33
A2	-1.32
A3	-2.61
A4	-9.9
A5	-10.87
A6	-13.83

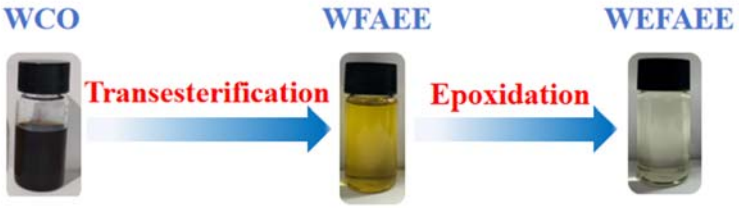

Figure S1. Photograph of WCO, WFAEE and WEFAEE showing the difference in their colors.

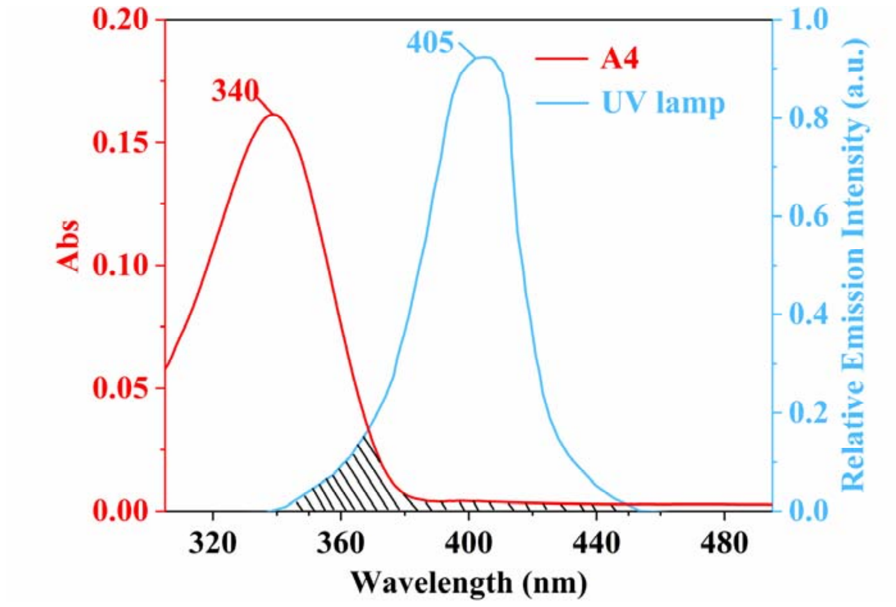

Figure S2. The absorption spectrum of the WMFAEE-HPA elastomer (A4 sample, red) and the emission spectrum of the LCD curing light sources (blue).

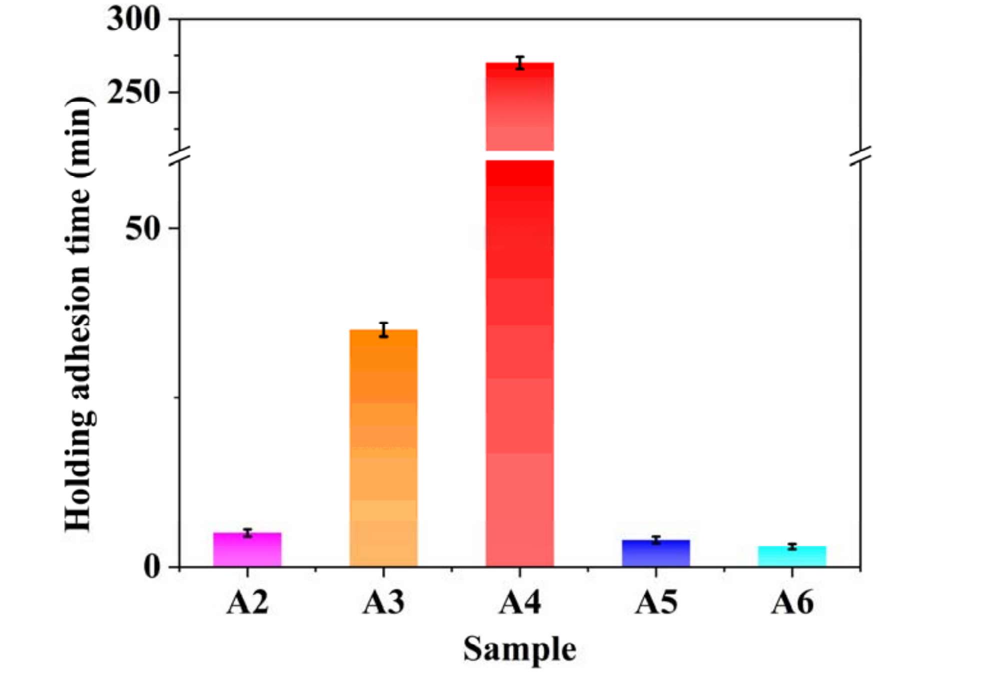

Figure S3. The holding adhesion times of WMFAEE-HPA elastomers on steel surface with different amounts of HPA.

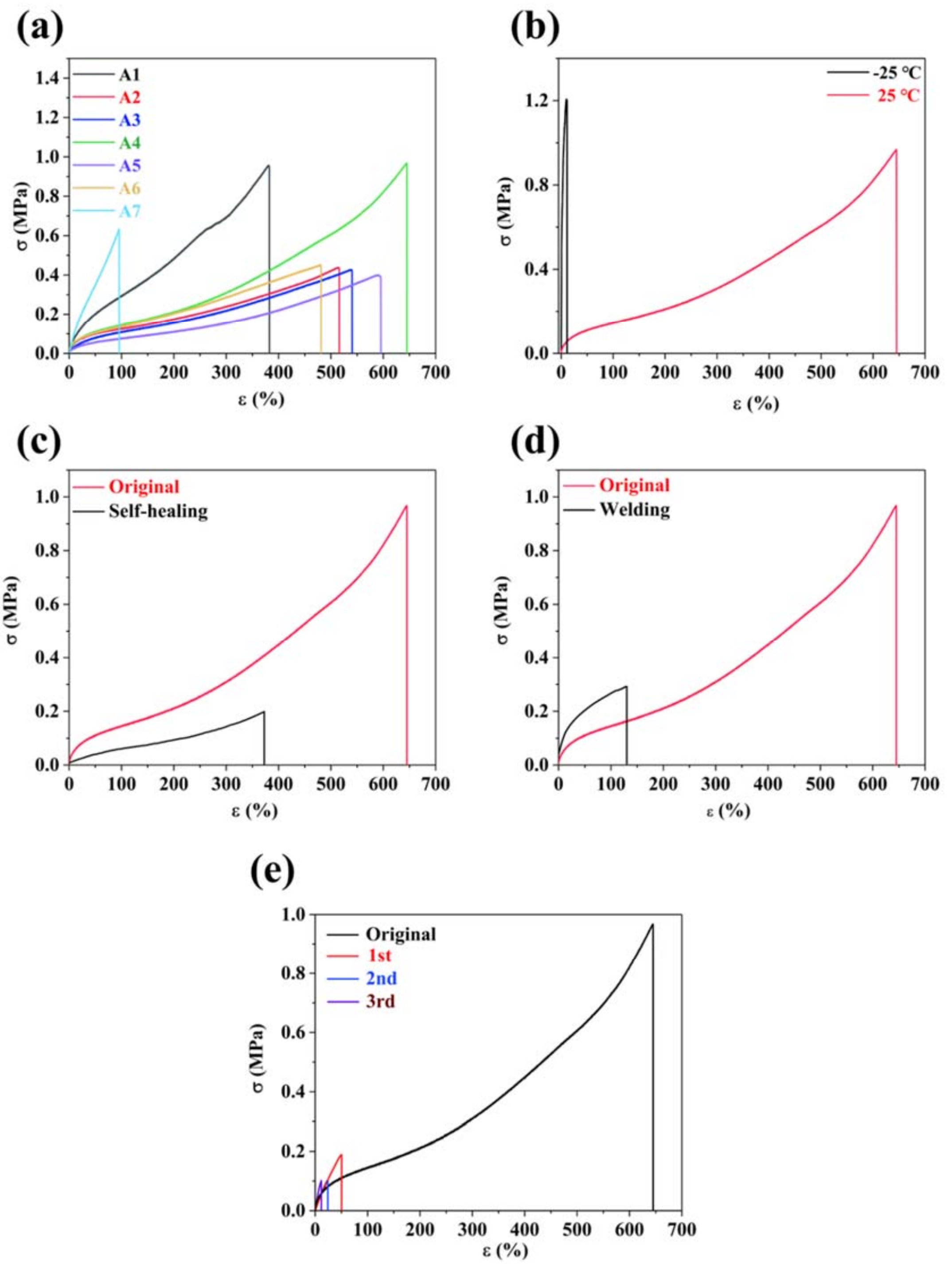

Figure S4. The stress-strain curves: (a) WMFAEE-HPA elastomers with varying HPA content; The pure HPA (A1) and EWOMA-HPA (A7) were both listed as control samples; (b) the A4 elastomer at different temperatures; (c) A4 elastomer before and after 24-hours self-healing; (d) A4 elastomer before and after 12-hours welding; (e) A4 elastomer before and after different reprocessing cycles.

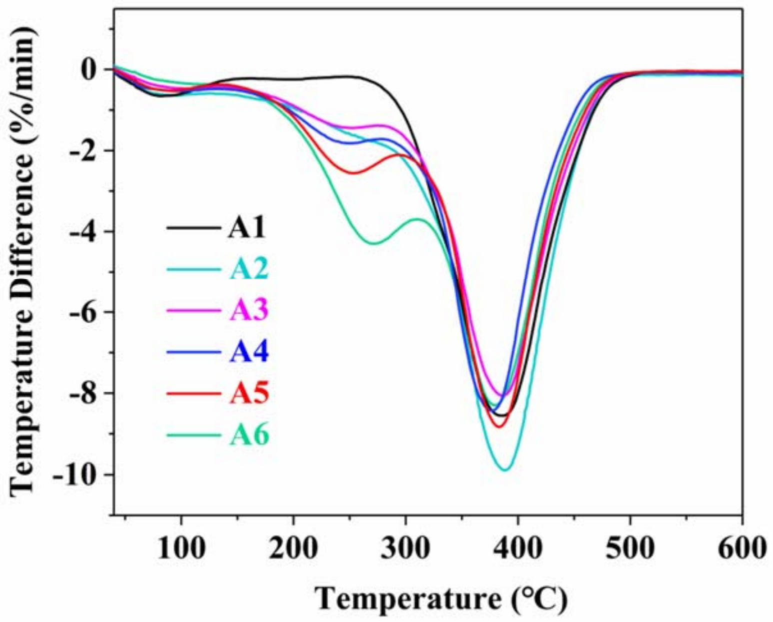

Figure S5. The DTG curves of WMFAEE-HPA elastomers and pure HPA (A1 sample).

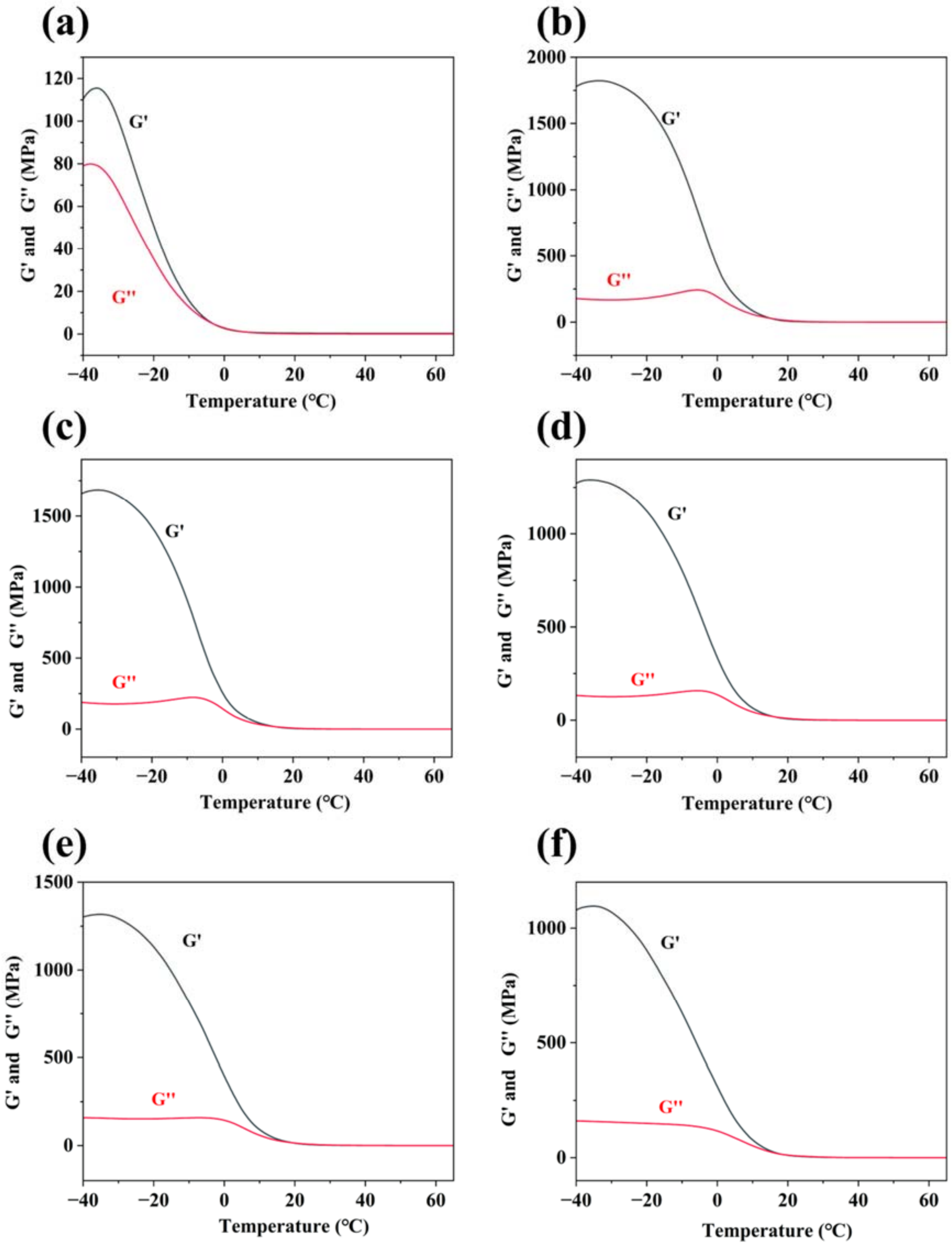

Figure S6. The DMA spectra of of WMFAEE-HPA elastomers and pure HPA (A1 sample).

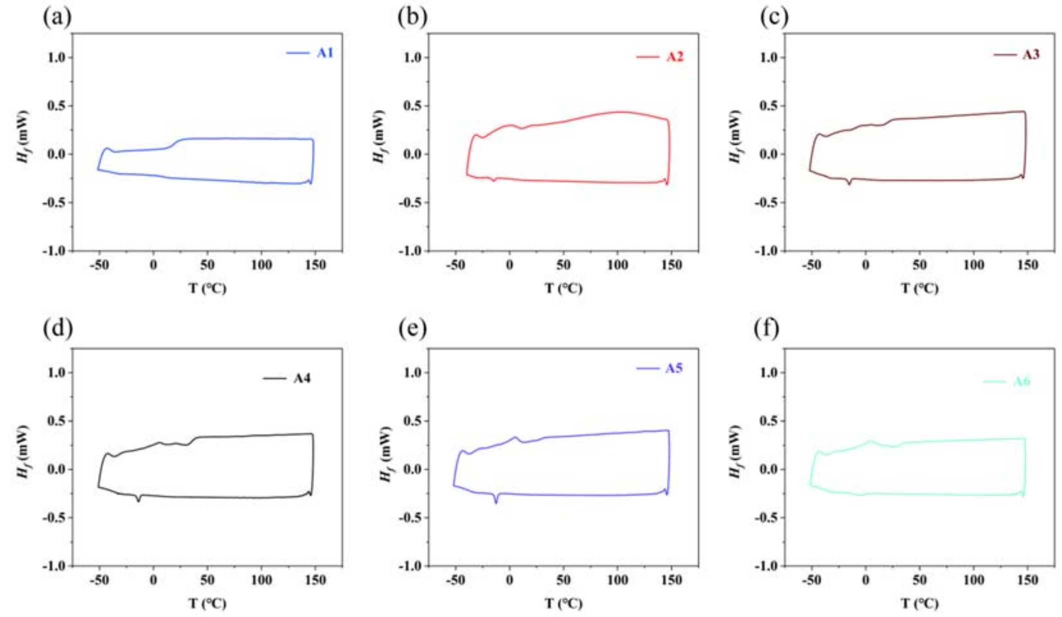

Figure S7. The DSC curves of of WMFAEE-HPA elastomers and pure HPA (A1 sample): (a) A1, (b) A2, (c) A3, (d) A4, (e) A5, (f) A6

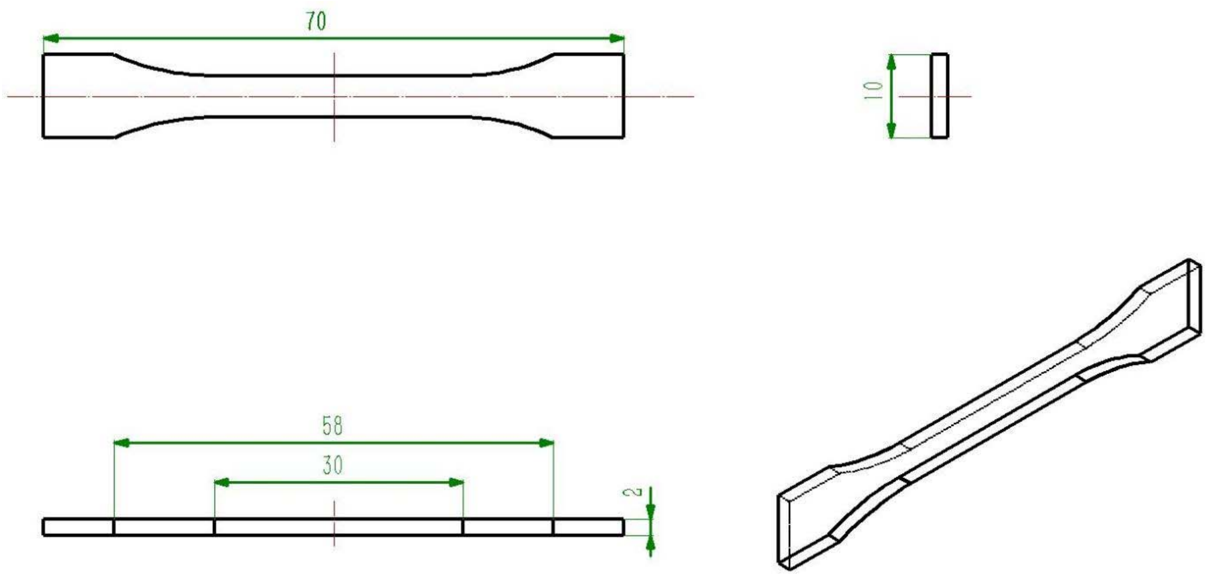

Figure S8. CAD model diagram of 1BA dumbbell-shaped spline according to the China Standard GB/T 1040.2-2006.

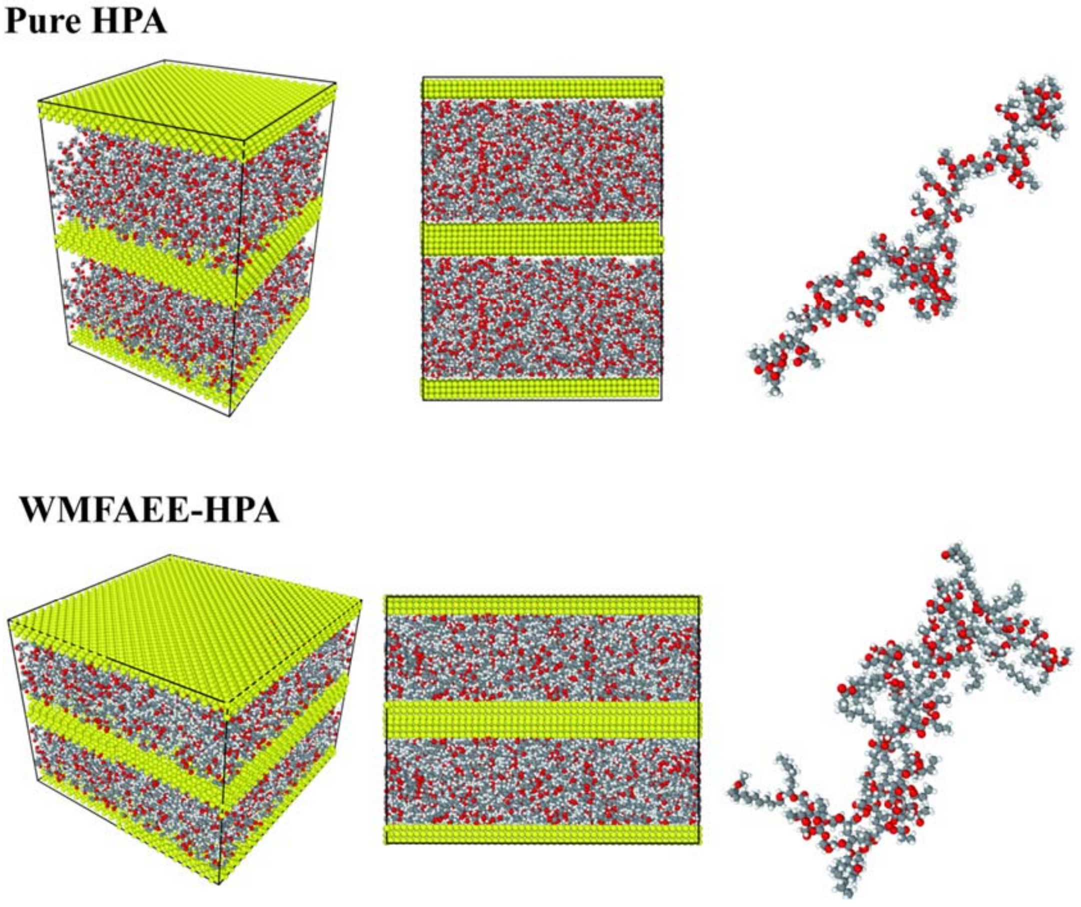

Figure S9. Modelling of HPA and WMFAEE-HPA with Cu matrix as interface before self-healing.

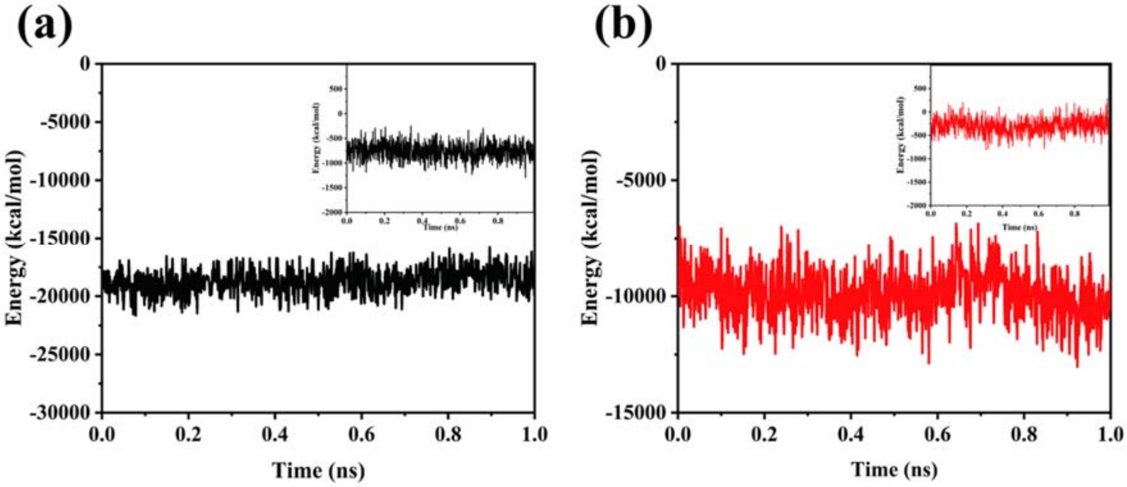

Figure S10. The cohesive energy of HPA (a) and WMFAEE-HPA (b), of which the insets are the cohesive energy of the single chain.
